# Public trust and global biobank networks

**DOI:** 10.1186/s12910-020-00515-0

**Published:** 2020-08-15

**Authors:** Lisa Dive, Christine Critchley, Margaret Otlowski, Paul Mason, Miriam Wiersma, Edwina Light, Cameron Stewart, Ian Kerridge, Wendy Lipworth

**Affiliations:** 1 Sydney Health Ethics, University of Sydney, Sydney, Australia; 2 Department of Psychological Sciences, School of Health Sciences, Swinburne University of Technology; and Centre for Law and Genetics, University of Tasmania, Hobart, Australia; 3 Faculty of Law, University of Tasmania, Hobart, Australia; 4 Taronga Institute of Science and Learning, Taronga Conservation Society, Mosman, Australia

**Keywords:** Biobanks, Trust, Globalisation, Commercialisation

## Abstract

**Background:**

Biobanks provide an important foundation for genomic and personalised medicine. In order to enhance their scientific power and scope, they are increasingly becoming part of national or international networks. Public trust is essential in fostering public engagement, encouraging donation to, and facilitating public funding for biobanks. Globalisation and networking of biobanking may challenge this trust.

**Methods:**

We report the results of an Australian study examining public attitudes to the networking and globalisation of biobanks. The study used quantitative and qualitative methods in conjunction with bioethical analysis in order to determine factors that may contribute to, and threaten, trust.

**Results:**

Our results indicate a generally high level of trust in biobanks and in medical research more broadly. Key factors that can reduce perceived trustworthiness of biobanks are commercialisation and involvement in global networking.

**Conclusions:**

We conclude that robust ethical oversight and governance standards can both promote trust in global biobanking and ensure that this trust is warranted.

## Background

Biobanks, which can be defined as structured collections of biological samples and associated data stored for the purposes of present and future research [15], provide an important foundation for genomic and personalised medicine. Advances in computational and information technology and data linkage platforms have greatly enhanced the potential of biobanks to identify biomarkers and develop new treatments.

Typically, biobanks have been housed within, or associated with, academic medical research institutions, governments, or commercial organisations (such as biotechnology or pharmaceutical companies). Increasingly, however, biobanks are becoming part of national or international networks, which greatly enhances their scientific power and scope and therefore their capacity to produce beneficial results.

A number of factors influence the sustainability of biobanks and biobank networks. Key among these is the degree to which potential participants trust those who oversee biobanks to protect their interests. Empirical and theoretical research has demonstrated the importance of public trust in fostering public engagement with biobanks, encouraging donation to biobanks, and facilitating public funding for biobanks [3, 5, 8, 13, 16, 17].

While there is no single definition of trust, it is generally understood as an expectation that a person will honour an explicit or implicit commitment. Rather than seeking a simple definition, most trust researchers operationalise trust. For example O’Neill, in assessing the conditions in which trust is placed, considers that trusting involves a belief in another person’s or institution’s reliability, and our expectation that they are likely to perform in a particular way – in other words, that they will be trustworthy [14]. Trustworthiness, in turn, relates to the perceived competence of a person or institution and their commitment to follow through on what they are trusted to do. Trust, by its nature, can only be granted by the trustor, it cannot be demanded by the party that wishes to be trusted [10].

In the context of global biobanking, the distinction between trust in individuals and trust in institutions is important. A biobank donor might, for example, have a high degree of interpersonal trust in the individual who takes their blood sample or a doctor who requests that they donate a tissue sample for research purposes. While there is evidence that individuals trust their own institutions more than they do others [6], and that trust in the individual “agent” of a biobank is strongly associated with that institution [4], it is not obvious that this extends to organisations that form networks with other organisations. An analysis of trust in the context of biobanking networking needs, therefore, to be cognisant of the differences between interpersonal trust and institutional or social trust – namely the kind of trust that we have in unknown groups of people (such as a profession) or organisations – and needs to elucidate the factors that increase or decrease trust in both individual biobankers and in global biobank networks [9].

In order to make sense of the shift from interpersonal to institutional trust one needs to understand the role of transitivity. Transitivity refers to the ability of a relationship between two parties to transfer to a third party with whom one of the others stands in a particular relation. In terms of trust, if person A trusts (person or institution) B, then transitivity concerns the question of the conditions under which A’s trust in B could extend to (person or institution) C with which B has a relationship.

Hardin explores how we can make the shift from individual-to-group or individual-to-institution trust, which is a crucial transition when considering trust in biobanks [9]. Hardin claims that in order to understand the trust people have in government (or other institutions), we need first to understand the trustworthiness of the agents of the institution; secondly, we need to account for the knowledge that the individual who might engage with that institution has of its trustworthiness.

The trustworthiness of a biobank is, therefore, determined in large part by the perceived trustworthiness of the representative of the biobank – whether a clinician, scientist, or laboratory technician. This individual’s trustworthiness is, in turn, grounded in their professional attributes and behaviours, as well as the credentials of the institution that they represent. The process of seeking consent from biobank donors for example, can be an important way that the trustworthiness of the institution is communicated to the donor.

When we consider how samples and data are shared in the context of biobank networks – and particularly global networks – the issue of transitivity becomes even more important. In this context, ongoing trust depends on the degree to which a person’s trust in the biobank to which they donated “travels” with their sample and/or data if it is shared with other biobanks. In addition to individual-to-institution transitivity, networking also makes salient the transitivity of trust between institutions.

A previous study of Australian biobank donors found that study participants were willing to cede some privacy and control over their sample and/or data, as long as they trusted the collecting biobank [3]. Research from the United States (U.S.) suggests that trust in biomedical research is associated with lower levels of privacy concerns, and that higher levels of trust are inversely related to preferences for notification about future use of specimens [20]. U.S. research also suggests that people are more likely to support sharing their genomic data with U.S. researchers, and that support for data sharing diminishes in relation to non-U.S. researchers [11]. Extending this work, we have conducted an empirical study into the ethics and politics of global biobank networks, exploring attitudes towards global biobank networking.

Here we present results from two empirical phases of the project: a national survey and in-depth interviews. We focus on the results related to willingness to donate to a biobank, and preferences about allowing broad consent for future use of samples. We take both of these to be indicators of trust, based on both a conceptual rationale (stemming from definitions of trustworthiness) and previous empirical research demonstrating that willingness to donate, consent, and trust are connected [3, 20]. There are also other possible explanations for willingness to donate and to allow a broad, open-ended consent, such as familiarity and altruism, that might not be reducible to trust. However, it seems likely that trust is a major explanatory concept. Willingness to donate was assessed in the survey, and attitudes towards trust were explored in greater depth in interviews, both with participants who were generally willing to donate, and also those who were generally opposed to donating samples to biobanks. To assess transitivity of attitudes that indicate trust, we assessed the extent to which they shift when moving from the Australian to a global context.

## Method

This study was conducted in Australia between 2017 and 2018. The measures and sample were part of a larger study designed to assess a wide range of issues associated with the globalisation of biobanks. Here we present the results of two phases of the project: a computer assisted telephone interview (CATI) survey and follow-up semi-structured interviews with members of the Australian public. Ethics approval for the CATI survey and interviews were granted by Swinburne University and The University of Sydney ethics committees respectively.

### Biobank survey

A total of 750 Australians over the age of 18 years who could speak English participated in the CATI survey which ran for 3 weeks in November 2017. Telephone numbers (61.3% mobile, 38.7% landline) were randomly generated by *Samplworx,* and were designed to represent the proportion of residents residing in each Australian State and Territory. The margin of error was 3.5% at the 95% confidence interval where responses are assumed to be evenly split (i.e., 50%), and response rates according to the American Association for Public Opinion Research (AAPOR)‘s (2009) definitions and calculations (i.e., RR1 – RR4) ranged from 6.0–9.0% with a cooperation rate of between 13.8–15.4%. The sample was representative of the Australian population in relation to state and territory, gender (52.4% women) and employment status, but was overrepresented by older people (M = 50.85, SD = 18.01, Range = 18–91) and those with a university education (48.3%). 82.1% described their ethnicity as Australian.

The survey consisted of 48 forced choice questions, meaning that participants were required to choose an option out of the available responses for each question. The questions were designed to explore issues relating to participation in and support for Australian and international biobanks including: overall willingness to donate/participate, identifiability of samples, ethics, consent preferences, participant engagement, withdrawal, funding, payment for participation, perceived benefits and governance. The survey is provided in Additional file 1; here we outline the questions of greatest relevance to trust.

Prior to completing the CATI survey, all survey respondents were read a definition of biobanks.^1^ This was important given that only 26.4% indicated that they “know enough about medical research involving biobanks?” (71.3% answered ‘No’ and 2.3% ‘Unsure’).

*Willingness to donate/participate*: immediately after the definition was read, survey respondents were asked whether they would be willing to participate in four biobanks with varying levels of globalisation:.

1. A biobank that was located entirely within Australia and used only by Australian researchers
2. An Australian biobank that allows its samples to be used by researchers located overseas
3. An Australian biobank that sent some of its samples to be stored in a biobank overseas
4. A biobank located overseas (i.e. a bank that is located entirely overseas and used by researchers located overseas)

To avoid order effects, the order of the four responses was randomly presented throughout the survey.

*Willingness to store tissue according to identifiability*: six questions were designed to assess respondents’ willingness to allow samples stored at three different levels of identifiability (coded, anonymous and identifiable) to be stored and used by either Australian or foreign biobanks. The three different sample categories were defined to all participants as follows (and in order of presentation):

#### Coded

‘Biological samples (like DNA, blood, cancer specimens and so forth) are usually given a code so that researchers do not know who you are, but can link back to other information about you for research purposes. While protections are put in place, a small risk of loss of confidentiality still remains.’

#### Anonymous

‘Biobanks can also store samples anonymously. Anonymous samples are samples that have been de-identified so that no one is able to work out who had donated the sample.’

#### Identifiable

‘Sometimes biological samples can be completely identifiable (i.e. the researchers and anyone else who accesses the sample would know who you are).’

After each definition, respondents were asked two questions, “Would you be willing to have a coded/anonymous/identifiable sample stored and used by an Australian biobank” and “Would you be willing to have a coded/anonymous/identifiable sample used and stored by biobank researchers located overseas”. The response options were ‘Yes’, ‘No’ or ‘Unsure’, and the order of the two questions was randomly presented.

The questions were designed to determine how the variation of factors such as biobank location and identifiability of samples affected willingness to donate. The impact of commercialisation of biobanks was also assessed (see Additional file 1).

*Consent preferences*: participants were asked six questions about their preferences for consent under different conditions, Two questions related to oversight by Human Research Ethics Committees, and the other four questions asked participants for their consent preferences in different scenarios (see Additional file 1)). The kinds of permission or consent were defined for all participants as follows, including the option of refusal of consent:

#### None

‘I would not give consent for my sample to be used for any research study.’

#### Specific consent

‘I would like to be asked permission before each new study that uses my tissue.’

#### Conditional consent

‘I would want to give consent on the basis that it was used for certain studies and not others.’

#### Broad consent

‘I would want to give consent so that my sample could be used for any project.’

Survey respondents were asked what kind of consent they preferred, depending on whether they were donating tissue to an Australian biobank used only by Australian researchers, an Australian biobank that allows its samples to be used by overseas researchers (with samples stored either in Australia or overseas), and if they were donating to a foreign biobank. They were also asked about their consent preferences depending on whether or not future proposed research using their sample would be approved by a Human Research Ethics Committee.

### Qualitative interviews

In order to gain a more comprehensive and nuanced understanding of the attitudes and beliefs that motivated participants’ responses to the survey, semi-structured interviews were conducted with 16 participants recruited from the survey. These 16 participants were drawn from a random subset of the CATI survey categorised into four groups according to their age and willingness to donate to an international biobank: Younger supporters (YS), Older supporters (OS), Younger opposers (YO) and Older opposers (OO). Participants were identified as either “supporters” or “opposers” based on their responses to four key questions about their willingness to donate to a biobank and for their sample to be used in biobank research under various conditions. More detail about the criteria for assigning respondents into these groups is in Additional file 1. Four people were interviewed from each sub-group.

An interview schedule was developed in consultation with expert members of the research team, drawing upon topics covered in the CATI survey—including participants’ willingness to donate to local and international biobanks, attitudes towards the storage and sharing of samples and data, as well as preferences for different kinds of permission or consent. In addition to the four types of consent described in the survey, qualitative interviews introduced the concept of dynamic consent, which uses a technology platform to allow donors to change their consent preferences over time.

Two members of the research team (MW and LD) were responsible for recruiting participants drawn from a random sample of survey respondents across different categories, and conducting the interviews. Interviews took place between May and July 2018, approximately 8 months after the CATI survey, and lasted between 20 and 45 min. All interviews took place via telephone, and verbal or written consent was obtained from all participants prior to the interview. All interviews were digitally recorded and transcribed.

Transcripts were entered into NVIVO 11 and were coded by MW and LD. The coding procedure was informed by Morse’s outline of the cognitive basis of qualitative research, [12] and Charmaz’s outline of data analysis in grounded theory [1]. This process involved initial line-by-line coding and gerunding to encode process and action. The codes were then synthesised into categories, and subsequently abstracted into concepts. A process of constant comparison was applied throughout the data analysis, with codes, categories and concepts refined and reorganised, as new codes, categories and concepts emerged.

## Results

### Willingness to donate

Close to 94% of survey respondents indicated they would be willing to donate a sample to an Australian biobank (see Table 1), with interviews similarly reflecting a highly positive attitude towards medical research:

“I’m very much in favour of all types of scientific research … that’s the most important thing.” [OS].

**Table 1:** Frequency And Percentage Of Responses For Willingness To Donate And Identification Of Samples

Question	Yes	No	Unsure
Willingness to donate			
A biobank that was located entirely within Australia and used only by Australian researchers	706	31	13
	94.1%	4.1%	1.7%
An Australian biobank that allows its samples to be used by researchers located overseas	619	107	24
	82.5%	14.3%	3.2%
An Australian biobank that sent some of its samples to be stored in a biobank overseas	606	123	21
	80.8%	16.4%	2.8%
A biobank located overseas (i.e. a bank that is located entirely overseas and used by researchers located overseas)	469	244	37
	62.5%	32.5%	4.9%
Identification of samples			
Coded samples - Stored and used by an Australian biobank	656	72	22
	87.5%	9.6%	2.9%
Coded samples - Used or stored by biobank researchers located overseas	448	266	36
	59.7%	35.5%	4.8%
Anonymous Samples - Stored and used by an Australian biobank	706	34	10
	94.1%	4.5%	1.3%
Anonymous Samples - Used or stored by biobank researchers located overseas	570	161	19
	76.0%	21.5%	2.5%
Identifiable Sample - Stored and used by an Australian biobank	494	217	39
	65.9%	28.9%	5.2%
Identifiable Sample - Used or stored by biobank researchers located overseas	314	401	35
	41.9%	53.5%	4.7%

Two significant variables found in the survey to influence willingness to donate to, biobanks were: 1) the location of the biobank (within Australia as opposed to overseas), and 2) whether the research was being conducted by a commercial entity as opposed to a not-for-profit institution.

While 94% of survey respondents would consider donating tissue to a biobank located in Australia and used only by Australian researchers, only 63% of respondents would consider donating tissue to a biobank located overseas, with 33% saying they would not consider donating to an overseas biobank (see Table 1).

Survey respondents were also significantly more willing to donate to a biobank that was government funded (86%) or part of a public research institution (94%), as compared to one funded by or associated with a pharmaceutical company (57%) or a biotechnology firm (59%).

Interviews similarly revealed concerns about profit-motivated research whether conducted in Australia or abroad:

“I wouldn’t want an item to be donated for the purpose of … benefit from a commercial point of view.” [YO].

“I’d probably be more inclined or trusting something like a university or a not-for-profit.” [YO].

“I dislike the idea of other countries mooching off us and then making a profit.” [OO].

However, for many participants the concerns about commercialisation were less persuasive than a desire to support medical research:

“I’d probably be okay with both as long as the purpose is kind of still good.” [YO].

“I do have a preference for the not-for-profit using the tissues, but if it gets to the point where a commercial medical research company needs or uses tissues for research and their research benefits me and let’s say people who have my health condition, then that’s okay as well.” [YS].

Governance and ethical standards for research were also factors that had a significant influence on willingness to donate. There was a preference among 76% of participants that samples donated to an overseas biobank would be handled, stored and used according to Australian ethical standards and governance procedures. Willingness to donate to an overseas biobank was higher if the biobank operated under the standards of a multinational organisation (66%) as compared to the standards of a foreign government (35%). This suggests that participants view the ethical standards developed by a number of countries working together as more trustworthy than a single foreign government, although Australian standards were perceived as the most trustworthy.

### Control over samples and data

Survey respondents tended to be permissive when it came to types of consent to both Australian and global biobanks, with broad consent being the most preferred form of consent. Many interview respondents did not express strong preferences to retain control over their samples, with participants commenting that: “I’d be happy with a broad blanket consent for any kind of future research” [YS], and “you can use my DNA for anything really” [YS]. Qualitative interviews that discussed dynamic consent did not have a significant impact on consent preferences.

Appropriate ethical oversight of future research involving their sample and/or data was an important concern for many respondents and a prerequisite for broader forms of consent. A clear majority (85%) of survey participants wanted future research projects using their tissue sample to be approved by an ethics committee regardless of whether the biobank was located in Australia or overseas.^2^ Interviews similarly pointed to the need for appropriate ethical oversight—particularly when consent is open-ended and where tissue was shared with overseas biobanks.

“I would like to know that it was a fairly rigorous protocol that was in place.” [OS].

“I would need to know that all the protocols were absolutely spot [on] if you like, before I would like it to go to another country.” [OS].

“If it’s going to an approved biobank … it’s going to be done properly.” [YO].

However, respondents were less willing to support broad consent to the use of tissue or data by an overseas (36%) compared to Australian (53%) biobanks (see Table 2 for further details of consent preferences), and some interview participants expressed reluctance about losing control over their samples or data if they were sent overseas:

“I wouldn’t want to just let it go and not have any control over what happens to it.” [YO].

**Table 2:** Frequency And Percentage Of Responses For Consent Options

Type of biobank	None	Specific consent	Cond. consent	Broad consent	Unsure
An Australian biobank that is used only by Australian researchers	28	141	185	392	4
	3.7%	18.8%	24.7%	52.3%	0.5%
An Australian biobank that allows its samples to be used by overseas researchers	87	168	190	301	4
	11.6%	22.4%	25.3%	40.1%	0.5%
An Australian biobank that stored its samples overseas and allowed these samples to be used by researchers located overseas	95	167	191	289	8
	12.7%	22.3%	25.5%	38.5%	1.1%
A foreign or overseas biobank	131	173	165	267	14
	17.5%	23.1%	22.0%	35.6%	1.9%

### Identifiability of samples

The survey revealed that identifiability of samples influenced how willing people were to have their sample and/or data stored by an Australian or overseas biobank. If samples were identifiable, 66% of respondents would agree to donate a biological sample for storage and use by an Australian biobank. However, only 42% would donate an identifiable sample for storage or use by a biobank located overseas. Qualitative interviews also suggested that some participants would be uncomfortable with their identifiable data being shared with other biobanks:

“… just the privacy thing …” [YS].

“… want it anonymous …” [YS].

There was a similar disparity for anonymous^3^ samples, with almost all respondents (94%) being willing to have an anonymous sample used and stored by an Australian biobank, compared to 76% being willing to provide an anonymous sample for use or storage overseas. For de-identified samples, 60% of respondents would allow a coded sample to be used or stored overseas, compared to 88% being willing to have a coded sample stored and used by an Australian biobank.

Particularly when it came to overseas biobanks, many people preferred their sample to be fully anonymised^3^:

“… don’t really want my name put on these sort of things …” [YS].

“I just want to give my sample and be done with it.” [YS].

## Discussion

This study is consistent with most other research in this area in that we found high levels of support for research and trust in the enterprise of biobank-based research [2, 18], and also higher levels of trust in public compared to private biobanks [3]. We found that the predominant aspects of biobank practice that influence public trust are lack of commercial involvement (supporting earlier findings), and location within Australia. This was evident in a higher willingness to donate; a preference for more open-ended consent (where the researcher or institution is entrusted to use the material in ways that are acceptable to the donor); and less concern about potential re-identification (which suggests the belief that personal information will be safeguarded and used appropriately). Furthermore, we found that ethical oversight of future research is important for ensuring the transitivity of trust.

Since trust is essential to the enterprise of biobank-based research, the lower levels of trust that the public appears to have in global biobank networks and in commercialisation of biobanking present important challenges to the expansion and globalisation of biobank-based research. In this regard, those involved in biobanking need to be alert to both the fragility of trust [19], and the fact that trust is something that cannot be demanded, but must be earned and conferred willingly by the trustor [10]. The fact that ethical oversight and standards of governance had a strong influence on participants’ willingness to donate—whether to local or international biobanks—suggests that, with the right kind of governance, it may well be possible to maintain and enhance trust. Visibility of ethical oversight and clear communication of governance standards becomes an important way that biobanks can demonstrate their trustworthiness to potential donors, and to maximise the potential for transitivity of that trustworthiness.

In this regard, it is particularly important for biobanks engaging in global networking to consider how their networking arrangements might influence the transitivity of trust. We observed a clear preference for Australian ethical standards and governance procedures to be applied even to international biobanks, as well as a belief that several countries working together to develop harmonised standards for ethical governance is preferable to any individual foreign country’s standards. Governance arrangements that explicitly ensure that Australian ethical standards are upheld by all researchers who might have access to samples or data via the global network may also be an important way that individual biobanks can demonstrate their trustworthiness.

A potential limitation of our study is we did not measure trust directly, but rather made an inference from measures of willingness to donate to a biobank under different conditions. While strong connections between trust and willingness have been established [3, 20], they are conceptually distinct. Trust was however explicitly explored in the qualitative interviews, and this confirmed its close relationship to willingness to donate, to provide broad consent and to generally positive attitudes towards global biobanks. Another limitation is that participants were on average older and more highly educated (with a greater proportion of university educated people) when compared to the general population. As such, the views and attitudes expressed in this study may not generalise to the wider Australian public. Both quantitative and qualitative phases were potentially affected by social desirability bias, although efforts to address this were made in the interviews, by stratifying participants into supporters and opposers.

Given that appropriate ethical oversight of future research using participants’ sample and/or data is an important factor in determining whether trust will be sustained, careful attention needs to be paid not only to overall models of governance but also to specific processes, such as consent. This is because the consent process is not only about obtaining permission to take a sample, but also allows the donor to express their values and preferences with respect to how their sample will be used, albeit to varying degrees depending on whether consent sought is broad or specific. Trustworthiness can be therefore be demonstrated by communicating to donors explicitly that – even in the context of global networking or the involvement of commercial entities – their sample and data will be used in ways that align with their preferences and values regarding research and the use of their tissue.

Clear material transfer agreements and other procedural mechanisms that operationalise biobank networking might also go some way towards addressing concerns about globalisation and commercialisation, if they are communicated in an accessible way to the public. Such agreements can help to make it transparent that biobanks are cognisant of the risks of globalisation and commercialisation and that research will be conducted with a high level of ethical oversight. Developing such mechanisms and communicating them in an appropriate way with a view to fostering the transitivity of trust can help to build public trust in the practice of global biobank networking.

## Conclusion

Globalisation has transformed many aspects of biomedical research, including research using collections of tissue and data. While globalisation of biobanking has enormous scientific potential there are several practical measures that need to be taken by those seeking to contribute to or build biobank networks in order foster public trust and its transitivity. Rigorous ethical oversight that incorporates recognisable standards, robust systems for stakeholder engagement, meaningful consent processes, strategic and authentic communication, and attention to the just distribution of research benefits will both promote trust in global biobanking and ensure that this trust is warranted.

## Supplementary information

**Additional file 1: Appendix 1:** Biobanks Survey. **Appendix 2:** Selection of interview groups (describes the method by which survey respondents were allocated to groups for purposes of qualitative follow-up interviews)

## Data Availability

The datasets used and/or analysed during the current study are available from the corresponding author on reasonable request.

## Footnotes

1 The script for the definition of biobanks was: “I’d like to begin by giving you a little information about biobanks. Biobanks are collections of biological samples, such as DNA, blood, cancer specimens, etc. that are stored for multiple purposes. In some cases, people are asked to donate their samples for medical research. Biobanks once used to be localised in one place. Today, however, biobanks are going global, which means that tissue collected in one location may be stored and/or used by researchers all over the world.”

2 Our data showed that 92% of respondents would want ethics approval for future research conducted by an Australian biobank, and 88% want ethics approval for research conducted by an overseas biobank. Following analysis of the small group (*n* = 43) who wanted ethics approval for Australian but not for foreign biobank research we suspect this disparity is due to error and/or misunderstanding, since respondents in this group were significantly less likely to be willing to donate to overseas biobanks, including those operating under the standards of a foreign government.

3 Survey respondents were asked about having their sample stored anonymously. However, we acknowledge that in the genomic age it is now questionable whether true anonymisation is possible. See, for example Erlich, Shor, Pe’er, & Carmi [7].
