# Supplementary material for: Public trust and global biobank networks
Source: BMC Med Ethics. 2020 Aug 15;21:73. doi: 10.1186/s12910-020-00515-0 (PMC7429755; doi:10.1186/s12910-020-00515-0)
Supplement: Supplementary file 1 — Additional file 1: Appendix 1: Biobanks Survey. Appendix 2: Selection of interview groups (describes the method by which survey respondents were allocated to groups for purposes of qualitative follow-up interviews) [file 12910_2020_515_MOESM1_ESM.docx]

**Supplementary materials**

**Appendix 1: Biobanks Survey**

**INTRODUCTION**

Hello, my name is [name] and I'm calling from Swinburne University in Melbourne. We're not selling anything - we're conducting a survey in collaboration with the University of Sydney concerning people's attitudes towards donating biological samples, such as blood samples, for research purposes.

Your phone number has been randomly computer generated so we don’t know your identity.

The survey takes about 15 minutes. Would you like to be involved?

Are you 18 years of age or older?

1. Yes (Go to Privacy Statement)

0. No (Go to next question)

IF NO:

Is there someone else at home who is 18 years of age or older?

1. Yes (Go to next question)

0. No (Failed screen – Go to end)

IF YES:

May I speak with him or her?

1. Yes (Go back to Introduction for person over 18)

0. No (Failed screen – Go to end)

**PRIVACY STATEMENT**

Before we begin, I want to let you know that the information you provide is strictly confidential and we are not recording this interview. We have no record of your name and address and you can stop the interview at any time if you do not wish to continue. Results from this survey will only be used for research purposes and will be published in scientific journals. Only group information is published so that no one can be identified. If at any time during the survey, you're not sure, then just say unsure.

**SURVEY QUESTIONS**

**I’d like to begin by giving you a little information about biobanks.**

**Biobanks are collections of biological samples, such as DNA, blood, cancer specimens, that are stored for multiple purposes. In some cases, people are asked to donate their samples for medical research. Biobanks once used to be localised in one place. Today, however, biobanks are going global, which means that tissue collected in one location may be stored and/or used by researchers all over the world.**

**OVERALL WILLINGNESS**

1a. Hypothetically speaking, if asked, would you consider donating your tissue to a biobank that was located entirely within Australia and used only by Australian researchers?

1. Yes
2. it depends
3. No
4. unsure

1.b.i. If asked, would you consider donating your tissue to an Australian biobank that allows its samples to be used by researchers located overseas?

1. Yes
2. it depends
3. No
4. unsure

1.b.ii. If asked, would you consider donating your tissue to an Australian biobank that sent some of its samples to be stored in a biobank overseas?

1. Yes
2. it depends
3. No
4. unsure

1.c. If asked, would you consider donating your tissue to a biobank located overseas? (i.e. a bank that is located entirely overseas and used by researchers located overseas)?

1. Yes
2. it depends
3. No
4. unsure

**IDENTIFIABILITY**

**Biological samples (like DNA, blood, cancer specimens and so forth) are usually given a number (coded) so that researchers do not know who you are, but can link back to other information about you for research purposes. While protections are put in place, a small risk of loss of confidentiality remains.**

2a. Would you be willing to have a coded sample stored and used by an Australian biobank?

1. Yes
2. it depends
3. No
4. unsure

2b. Would you be willing to have a coded sample used or stored by biobank researchers located overseas?

1. Yes
2. it depends
3. No
4. unsure

**Biobanks can also store samples anonymously. Anonymous samples are samples that have been de-identified so that no one is able to work out who had donated the sample**

3a. Would you be willing to have an anonymous sample stored and used by an Australian biobank?

1. Yes
2. it depends
3. No
4. unsure

3b. Would you be willing to have an anonymous sample used or stored by biobank researchers located overseas?

1. Yes
2. it depends
3. No
4. unsure

**Sometimes biological samples can be completely identifiable (i.e. the researchers and anyone else who accesses the sample would know who you are).**

4a. Would you be willing to have an identifiable sample stored and used by a Australian biobank?

1. Yes
2. it depends
3. No
4. unsure

4b. Would you be willing to have an identifiable sample used or stored by biobank researchers located overseas?

1. Yes
2. it depends
3. No
4. unsure

**CONSENT AND INFORMATION**

Most biobanks are storage facilities that distribute tissues to many researchers for many different research projects. In most cases, this distribution is overseen by a Human Research Ethics Committee. This is a group comprised of scientific and ethical experts and laypeople who consider the rights and wellbeing of tissue donors in research.

5.ai. if you donated your tissue to an Australian biobank, would you expect that all research projects using your tissue had been approved by a human research ethics committee?

1. Yes
2. it depends
3. No
4. unsure

5.aii. if you donated your tissue to a foreign or overseas biobank, would you expect that all research projects using your tissue had been approved by a human research ethics committee?

1. Yes
2. it depends
3. No
4. unsure

We are now interested in your views about the kind of consent, or permission, you would want to give if you did donate your tissue in different scenarios. The first type of consent is “None”, where you would not give consent for your sample to be used for any research study. The second is “Specific consent” where you would like to be asked permission before each new study that uses your tissue. The third is “Conditional consent” where you would want to give consent for certain kinds of studies but not for others. The fourth option is “Broad consent” where you would consent just once for your sample to be used for any future projects that are approved by an ethics committee.

**I will now read the scenarios.**

5.b.i. If you donated your tissue to an Australian biobank that is used only by Australian researchers, what kind of consent would you prefer? [Interviewer : Only read out “None, Specific consent, conditional consent and broad consent”. Repeat consent type only if respondent asked]

1. None. [I would not give consent for my sample to be used for any research study].
2. Specific consent. [I would like to be asked permission before each new study that uses my tissue].
3. Conditional consent. [I would want to give consent for certain kinds of studies but not for others].
4. Broad consent. [I would want to give consent so that my sample could be used for any future project approved by an ethics committee].

5.b.ii. If you donated your tissue to an Australian biobank that allows its samples to be used by overseas researchers, what kind of consent would you prefer?

1. None. [I would not give consent for my sample to be used for any research study].
2. Specific consent. [I would like to be asked permission before each new study that uses my tissue].
3. Conditional consent. [I would want to give consent for certain kinds of studies but not for others].
4. Broad consent. [I would want to give consent so that my sample could be used for any future project approved by an ethics committee].

5.b.iii. If you donated your tissue to an Australian biobank that stored its samples overseas and allowed these samples to be used by researchers located overseas?

1. None. [I would not give consent for my sample to be used for any research study].
2. Specific consent. [I would like to be asked permission before each new study that uses my tissue].
3. Conditional consent. [I would want to give consent for certain kinds of studies but not for others].
4. Broad consent. [I would want to give consent so that my sample could be used for any future project approved by an ethics committee].

5.b.iii. If you donated your tissue to a foreign or overseas biobank, what kind of consent would you prefer? [Interviewer: if needed define foreign biobank i.e. a bank that is located entirely overseas and used by researchers located overseas]?

1. None. [I would not give consent for my sample to be used for any research study].
2. Specific consent. [I would like to be asked permission before each new study that uses my tissue].
3. Conditional consent. [I would want to give consent for certain kinds of studies but not for others].
4. Broad consent. [I would want to give consent so that my sample could be used for any future project approved by an ethics committee].

FEEDBACK

**In addition to asking people for their permission to use samples, researchers can also keep participants informed, for example via a website, about how their tissue is being used.**

6a. If your tissue was stored in an Australian biobank, please indicate by answering yes or no, what information would you like to be given?

1. Where the tissue is being stored [YES, NO, UNSURE]
2. When the tissue is being used [YES, NO, UNSURE]
3. The nationality of the researchers using your tissue [YES, NO, UNSURE]
4. What type of research it is being used in [YES, NO, UNSURE]
5. The general results of the research [YES, NO, UNSURE]
6. Results of the research, but only if they apply to me or my family [YES, NO, UNSURE]

6b. If your tissue was stored in a foreign biobank, what information would you like to be given about it (indicate as many as apply)?

1. Where the tissue is being stored [YES, NO, UNSURE]
2. When the tissue is being used [YES, NO, UNSURE]
3. The nationality of the researchers using your tissue [YES, NO, UNSURE]
4. What research it is being used in [YES, NO, UNSURE]
5. The general results of the research [YES, NO, UNSURE]
6. Results of the research, but only if they apply to me or my family [YES, NO, UNSURE]

**WITHDRAWAL**

**Sometimes when people donate tissue to a biobank they are given the option of being allowed to withdraw their tissue at any time in the future if they change their mind. For each of the following questions please respond with either Yes, It depends or no.**

6a. If you donated your tissue to an Australian biobank, would you want to have the ability to withdraw your donated tissue at any time?

1. Yes
2. it depends
3. No
4. unsure

6b. If you donated your tissue to an Australian biobank that sent some of its material overseas for storage in a foreign biobank, would you want the ability to withdraw your donated tissue at any time?

1. Yes
2. it depends
3. No
4. unsure

6c. If you donated your tissue to a foreign or overseas biobank, would you want the ability to withdraw your donated tissue at any time?

1. Yes
2. it depends
3. No
4. unsure

**COMMERCIALISATION**

Biobanks also differ in their purpose and the way that they are funded. Some are funded by government and are directed to public benefit while others are funded by private companies and are used to make new products for profit, and some are both.

Would you be willing to donate to the following types of biobanks? The response options are again, Yes, It depends and NO. [RANDOMISE QUESTIONS]

7a. an Australian biobank that operated as a not-for-profit organization?

1. Yes
2. it depends
3. No
4. unsure

7b. an overseas biobank that operated as a not-for-profit organization

1. Yes
2. it depends
3. No
4. unsure

7c. an Australian biobank that operated for profit

1. Yes
2. it depends
3. No
4. unsure

7d. an overseas biobank that operated for profit

1. Yes
2. it depends
3. No
4. unsure

8. If you donated your tissue sample to the following types of biobanks, please let us know whether you would expect to be reimbursed for your time and effort. The response options are again, Yes, It depends and NO. [RANDOMISE QUESTIONS]

8a. an Australian biobank that operated as a not-for-profit organization?

1. Yes
2. it depends
3. No
4. unsure

8b. an overseas biobank that operated as a not-for-profit organization

1. Yes
2. it depends
3. No
4. unsure

8c. an Australian biobank that operated for profit

1. Yes
2. it depends
3. No
4. unsure

8d. an overseas biobank that operated for profit

1. Yes
2. it depends
3. No
4. unsure

BENEFITS

We are now interested in what you think about the distribution of benefits that may arise from biobank research. Please respond again with either Yes, It depends or No.

9a. Would you want most of the benefits from research using samples from an Australian biobank to go to Australians?

1. Yes
2. it depends
3. No
4. Unsure

9b. Would you want benefits from research using samples from an Australian biobank to go to people from other countries?

1. Yes
2. it depends
3. No
4. unsure

9c. Would you donate your tissue to an overseas biobank if people living in poverty would not benefit?

1. Yes
2. it depends
3. No
4. unsure

9d. Would you personally expect to benefit from research done by an Australian biobank using your sample?

1. Yes
2. it depends
3. No
4. unsure

9e. Would you personally expect to benefit from research done by a foreign or overseas biobank using your sample?

1. Yes
2. it depends
3. No
4. unsure

9f. Would you personally expect to benefit from research on samples donated by people from other countries?

1. Yes
2. it depends
3. No
4. unsure

**GOVERNANCE**

**Biobanks in different countries operate under different codes and practices. Sometimes the differences are great, sometimes they are small.**

10a. If you donated a sample to a foreign or overseas biobank, would you expect your sample to be handled, stored and used according to Australian standards?

1. Yes
2. it depends
3. No
4. unsure

10b. Would you be willing to donate a sample to an overseas biobank if they operated under the standards of a foreign government?

1. Yes
2. it depends
3. No
4. unsure

10c. Would you be willing to donate a sample to an overseas foreign biobank if they operated under the standards of an organization with multiple member countries such as the European Union?

1. Yes
2. it depends
3. No
4. Unsure

10d. If you had a particular interest in a specific research project that was conducted by a biobank overseas, would you donate a sample to that biobank even if it was illegal to conduct that same research in Australia?

1. Yes
2. it depends
3. No
4. unsure

**PUBLICATION AND COMMUNICATION**

12. If you donated your tissue to a biobank, would you like it if there was a website through which you could give your consent, manage the use of your tissue and relevant medical information, and find out about the research that is being done?

1. Yes
2. it depends
3. No
4. unsure

13. Do you feel that you know enough about medical research involving biobanks?

1. Yes
2. it depends
3. No
4. unsure

14. If you have an email address, would you like us to let you know the summary of results from this study in due course?

1. Yes
2. it depends
3. No
4. unsure

15. Do we have your permission to contact you for future research into public attitudes towards biobanking?

1. Yes
2. it depends
3. No
4. unsure

**DEMOGRAPHICS**

"Q17. That's the end of the survey questions, but before we finish I need to ask you some demographic information please.

What is your current employment status?

Q17 Employment Status

1. Work full time

2. Work part time

3. Home duties

4. Retired

5. Unemployed

6. Other (Specify Q17a)

------------------

8. Unsure

9. Refused

Q18 Education

"Q18. What is the highest level of education you have _completed_?

1. Less than Year 12 Secondary School

2. Year 12 Secondary School

3. TAFE diploma or certificate

4. University degree or diploma

5. Postgraduate degree

--------------------------

8. Unsure

9. Refused

Q19 Church Attendance

"Q19. Apart from special religious ceremonies such as weddings and funerals, how often do you attend religious services?"

0. Never

1. Less than once a year

2. At least once a year

3. Several times a year

4. At least once a month

5. At least once a week

-----------------------

8. Unsure

9. Refused

Q20 Spiritual beliefs

"Q20. How important are spiritual beliefs to you"

1. Not at all important

2. Not very important

3. Quite important

4. Very important

----------------------------

8. Unsure

9. Refused

Q21 Ethnicity

"Q21. When asked what your nationality or ethnicity is, what do you usually say?"

1. Australian

2. Other (specify Q21a)

--------------------------------

8. Unsure

9. Refused

Q22 Year Born

"Q22. In what year were you born?

Q23 Gender

"Q23. _INTERVIEWER PROMPT: Record gender by voice. If unsure, read the question as follows:

This next question may seem a bit odd, but I'm obliged to ask if you are Male, Female or other?_"

1. Male

2. Female

Q24 Postcode

"Q24. May I have your postcode please?

MOBILE OR LANDLINE PHONE

Q25 FOR CALLS TO LANDLINES

"Q25. Do you have a mobile phone?"

1. Yes

2. No

------------------

9. Refused

FOR CALLS TO MOBILES

Q26 Have Land Line

"Q26. Do you have a Land Line phone?"

1. Yes

2. No

------------------

9. Refused

FOR THOSE WITH BOTH LAND LINE AND MOBILE

Q27 Phone used more

"Q27. Which type of phone do you use more often?"

1. Mobile

2. Land line

3. Use mobile and land line about the same

------------------------------------------------

8. Unsure

9. Refused

FOR CALLS TO MOBILES

Q28 Where mobile survey

"Q28 May I ask where you have been while we have been speaking?"

1. At home

2. At work

3. Visiting family / friend

4. Shopping Centre

5. Driving

6. Other (specify Q28a)

-----------------------------------

9. Refused

Q29HLOC Home LOC

"Q29HLOC. Which state / territory do you live in?

Only asked this question if calling a mobile sample.

Otherwise, location is just filled in "

1. NSW

2. ACT

3. NT

4. QLD

5. SA

6. TAS

7. VIC

8. WA

FOR PEOPLE WHO AGREED TO FOLLOW UP IN Q14 & Q15

Q30 Follow Up Contact

"Q30 Thank you very much for participating in this survey.

May I now take down some contact details so we can send you a summary of the result/contact you for future research

1. Yes ] Q31DETAILS

2. No ] Q99END

Q31TNAME

Q31PHONE

Q31EMAIL

Q31DETAILS

EDIT

"Q31DETAILS

Please be assured that your contact details will be recorded on a separate database from the responses you have just given and will not be matched to your survey responses in any way.

_INTERVIEWER PROMPT: Make sure you include the area code for landlines_

FIRST NAME: [Q31TNAME.........................]

PHONE: [Q31PHONE..........]

EMAIL: [Q31EMAIL......................................................]

"

Q99END

"Thank you very much for participating in this survey. We appreciate you giving your time, and your contribution has been very valuable. Would you like any further information about the project or a contact number for any complaints?

Christine Critchley

Department of Statistics, Data Science and Epidemiology

Swinburne University of Technology

PO Box 228

Hawthorn 3122

(03) 9214 5480

ccritchley@swin.edu.au"

If you have any concerns or complaints about the conduct of this project, please contact:

Research Ethics Officer

Office of Research & Graduate Studies

Swinburne University of Technology

PO Box 218

HAWTHORN VIC 3122

Tel (03) 9214 5218 or resethics@swin.edu.au

OK, that is the end of the survey. Thank you very much for your time_"

**Appendix 2: Selection of interview groups**

To ensure a range of views from those who would or would not donate their tissue to an international biobank two groups of participants were created from the survey responses: supporters and opposers. A total of 623 respondents who provided permission to be contacted for future research were classified as either supporters (n = 242) or opposers (n = 97) based on their responses to the following four questions:

1.c. If asked, would you consider donating your tissue to a biobank located overseas? (i.e. a bank that is located entirely overseas and used by researchers located overseas)?

2b. Would you be willing to have a coded sample used or stored by biobank researchers located overseas?

3b. Would you be willing to have an anonymous sample used or stored by biobank researchers located overseas?

4b. Would you be willing to have an identifiable sample used or stored by biobank researchers located overseas?

Supporters were defined as those who responded yes to all four questions, while opposers responded no to all. A median split for age (median = 53 years) was then used to separate all four groups into older and younger groups. Four lists of participants were created and were randomly selected to be interviewed until the required number was met.
